# Role of PCNA and TLS polymerases in D-loop extension during homologous recombination in humans^☆^

**DOI:** 10.1016/j.dnarep.2013.05.001

**Published:** 2013-09

**Authors:** Marek Sebesta, Peter Burkovics, Szilvia Juhasz, Sufang Zhang, Judit E. Szabo, Marietta Y.W.T. Lee, Lajos Haracska, Lumir Krejci

**Affiliations:** a National Centre for Biomolecular Research, Masaryk University, Brno, Czech Republic; b Department of Biology, Masaryk University, Brno, Czech Republic; c International Clinical Research Center, Center for Biomolecular and Cellular Engineering, St. Anne's University Hospital Brno, Brno, Czech Republic; d Institute of Genetics, Biological Research Center, Hungarian Academy of Sciences, Szeged, Hungary; e Department of Biochemistry and Molecular Biology, New York Medical College, Valhalla, NY, USA; f Institute of Enzymology, Research Centre for Natural Sciences, Hungarian Academy of Sciences, Budapest, Hungary

**Keywords:** TLS polymerases, Homologous recombination, DNA repair synthesis, D-loop, Reconstitution

## Abstract

Homologous recombination (HR) is essential for maintaining genomic integrity, which is challenged by a wide variety of potentially lethal DNA lesions. Regardless of the damage type, recombination is known to proceed by RAD51-mediated D-loop formation, followed by DNA repair synthesis. Nevertheless, the participating polymerases and extension mechanism are not well characterized. Here, we present a reconstitution of this step using purified human proteins. In addition to Pol δ, TLS polymerases, including Pol η and Pol κ, also can extend D-loops. In vivo characterization reveals that Pol η and Pol κ are involved in redundant pathways for HR. In addition, the presence of PCNA on the D-loop regulates the length of the extension tracks by recruiting various polymerases and might present a regulatory point for the various recombination outcomes.

## 1 Introduction

Homologous recombination (HR) is a crucial error-free pathway for repairing a diverse array of potentially lethal lesions. These lesions include interstrand crosslinks, ssDNA gaps left behind the replication fork, and double-strand DNA breaks (DSB) due to DNA damaging agents that cause replication to occur through damaged template or by programmed events (e.g., during meiosis) [1,2]. HR comprises a complex multistep pathway that requires formation of RAD51 nucleoprotein filament on ssDNA. This ssDNA is generated by nucleolytic processing of DSB or arises from perturbed replication. RAD51 filament then facilitates the formation of a physical connection between invading and homologous template DNA that leads to generation of a transient structure known as D-loop [3]. The 3′-OH end of an invading strand within this structure then serves as a primer for the replication machinery to copy the missing genetic information in an error-free manner. The pathway can proceed by two alternative mechanisms. In the double-strand break repair sub-pathway, the extended D-loop is stabilized by capturing the second end of the DSB and leads to formation of a double Holliday junction. The Holliday junctions can then be resolved to produce crossover and non-crossover products [4]. Alternatively, the extended invaded strand is released from the D-loop and anneals with the second end of the DSB. This pathway is referred to as the synthesis-dependent strand-annealing pathway and exclusively results in generation of non-crossovers [5]. While many of the HR steps have been studied in detail, only very little is known about the DNA synthesis step.

Both types of DNA polymerases, replicative as well as translesional (TLS), have been suggested to play a role in recombination-associated DNA synthesis [6–9]. While the highly accurate and processive polymerases δ and ɛ possess proofreading activity and are involved in genome replication, the TLS polymerases are specialized in synthesis across damaged bases. These polymerases lack proofreading activity and also show low processivity [10]. Processivity of the polymerases is enhanced by proliferative cell nuclear antigen (PCNA), a trimeric complex that encircles DNA and prevents DNA polymerases from dissociating from DNA and serves also as a docking site for a diverse set of factors involved in DNA replication and repair [11]. The association with these factors can also be regulated by post-translational modification of PCNA [12]. In response to DNA damage, PCNA becomes ubiquitinated at the conserved lysine K164, resulting in recruitment of the TLS polymerases [13]. Recently, a role also for PCNA SUMOylation has been proposed in human cells to control HR [14,15]. The roles of these individual factors in DNA repair extension are not clear. Genetic experiments performed in the yeast *Saccharomyces cerevisiae* point to involvement of the replicative polymerase δ as well as polymerase ζ in DNA recombination-associated synthesis [16–18]. However, studies in humans and chicken DT40 cells imply activity also of DNA polymerase η in this step of HR [8,9]. This is in agreement with in vitro experiments where the yeast polymerase δ, and, to a lesser extent also polymerase η, extended the plasmid-based D-loop substrate [19,20].

The present study aimed to understand the molecular mechanism of the DNA synthesis step of HR in humans. An in vitro system was used to examine the ability of different human polymerases to extend RAD51-mediated D-loop structure. We found that human polymerase δ, together with PCNA, efficiently extends D-loops, with the extension tracks reaching up to 2 kb. The TLS polymerases η, κ and ι were also tested for their abilities to extend recombination intermediates. Although less efficient compared to Pol δ, Pol η and Pol κ, they extended the D-loop substrate while generating shorter extension tracks. The involvement of TLS polymerases in HR was also corroborated in vivo, as their overexpression or down-regulation affected the recombination frequencies. We further demonstrate that polymerase η extends D-loops independently of PCNA, pointing to the possibility that the presence of PCNA may regulate the length of the extension tracks and thus alter the outcome of HR.

## 2 Materials and methods

### 2.1 Proteins and DNA substrate

A detailed description of protein purifications and the DNA substrate can be found in the supplementary material (Supplementary Fig. 1A).

### 2.2 D-loop and primer extension assay

The D-loop reaction was carried out essentially as described in Petukhova et al. [21] and Ptukhova et al. [22]. Radioactively labeled or unlabeled D1 oligonucleotide [21,22] (AAATCAATCTAAAGTATATATGAGTAAACTTGGTCTGACAGTTACCAATGCTTAATCAGTGAGGCACCTATCTCAGCGATCTGTCTATTT) (3 μM nucleotides) was incubated for 5 min at 37 °C with RAD51 (1 μM) in 10 μl of buffer R (35 mM Tris–Cl, pH 7.4, 1 mM ATP, 1.25 mM MgCl_2_, 50 mM KCl, 1 mM DTT and an ATP-regenerating system consisting of 20 mM creatine phosphate and 20 μg/ml creatine kinase). Then, 1 μl of Hop2-Mnd1 (200 nM) was added and the mixture incubated for an additional 1 min at 37 °C. The reaction was started by adding 1 μl of pBluescript SK(−) replicative form I (50 μM base pairs), and the mixture was incubated for 5 min at 37 °C.

RPA (660 nM), PCNA (30 nM) and RFC (10 nM) were then added in buffer O (20 mM Tris–Cl, pH 7.5, 5 mM DTT, 0.1 mM EDTA, 150 mM KCl, 40 μg/ml BSA, 8 mM MgCl_2_, 5% [v/v] glycerol, 0.5 mM ATP, and 100 μM each of dGTP, dCTP and dTTP) to a final volume of 30 μl. Concentrations are with respect to the final 30 μl reaction volume. If the reaction was monitored using D1 oligonucleotide, then 100 μM unlabeled dATP was used. When dATP incorporation was monitored, 0.375 μCi [α-^32^P]dATP and 25 μM dATP were used. The mixture was then incubated for 5 min at 30 °C. The DNA synthesis was started by the addition of corresponding polymerase (as indicated in the figures). After 10 min (Pol δ) or 20 min (Pol η, Pol κ) at 37 °C, the reactions were stopped by incubation at 37 °C for 3 min with SDS (0.5% final) and proteinase K (0.5 mg/ml) and then loaded onto an agarose gel (0.8%, w/v). After electrophoresis, the gel was dried on DE81 paper and exposed on a phosphorimager screen. Scanning and quantification of the results were done using a Fuji FLA 9000 imager, followed by analysis using MultiGauge software (Fuji).

### 2.3 2D gel electrophoresis

Electrophoresis was performed as described by Sebesta et al. [20]. D-loop formation and primer extension reactions were split into two parts and electrophoretically separated in 0.8% (w/v) agarose gel and 1× TAE buffer. The lanes were then excised from the gel. One lane displaying D-loops and products from aliquots of each reaction mixture was dried and the remainder of the gel was soaked for 60 min in denaturing buffer (50 mM NaOH, 1 mM EDTA). The gel was then loaded onto a denaturing agarose gel (1% [w/v] in 50 mM NaOH, 1 mM EDTA) and run for 6 h. The dried gel was exposed to a phosphorimager screen, visualized using a Fuji FLA 9000 imager, and then analyzed using MultiGauge software (Fuji).

### 2.4 Recombination assay

HeLa cells were cultured in DMEM supplemented with 10% FCS and antibiotics. Cells were transfected with Lipofectamine 2000 (Invitrogen) according to the manufacturer's instructions. A recombination reporter assay based upon green fluorescent protein (GFP) was used in the HeLa cells (Supplementary Fig. 2). It measures the recombination frequency between one copy of integrated DNA fragment encoding a C-terminally truncated GFP and a transiently transfected DNA fragment encoding N-terminally truncated GFP proteins. Briefly, reporter cells were transfected first with a silencing siRNA or a protein expression construct. After 48 h, cells were transfected again with siRNA and targeting vector encoding N-terminally truncated GFP proteins and I-SceI expression plasmid. One day after the second transfection, cells were selected for puromycin resistance and the frequency of GFP + recombinants was analyzed. The following siRNAs (purchased from Ambion) were used to down-regulate individual genes: XPF (GGA GGA GUA UUU UAU CAA Utt), XRCC4 (GCC GCU AUU ACC GUA UCU Utt), Pol η (CCA CGT CTC TGG AAT CAT Ttt), Pol κ (CAG TAG ATT GTA TAG CTT Ttt), and Pol ι (GGT GGT TAC CTG CAA CTA Ttt). For expression study, we generated N-terminally FLAG-tagged Pol η, Pol κ and Pol ι expressing plasmids using the pRK2F-based expression vector. The expression plasmids without tag for PARP1 and Rad51AP1 were purchased from OriGene (Rockville, MD, USA).

## 3 Results

### 3.1 Reconstitution of recombination-associated DNA synthesis in vitro

**Fig. 1:**
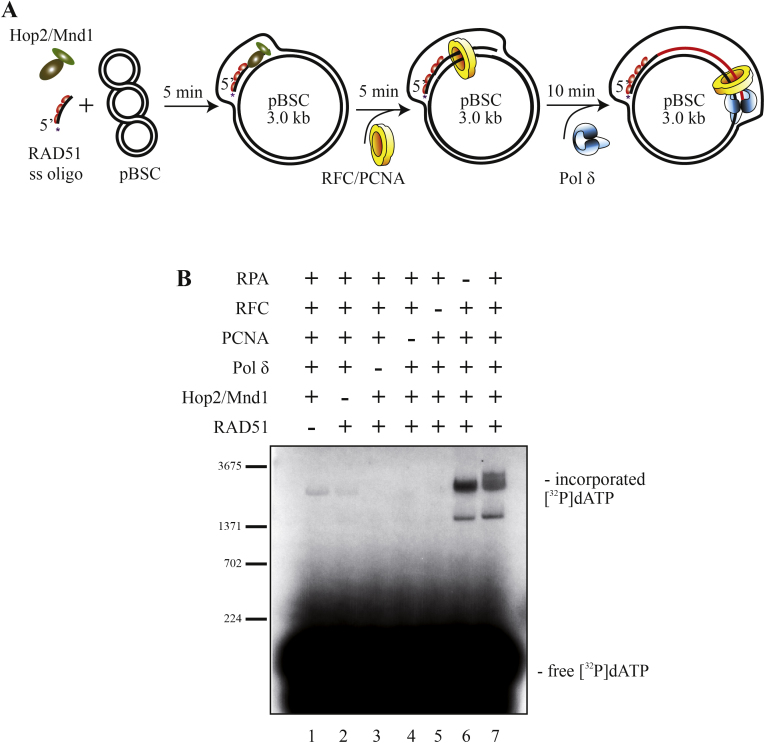
Reconstitution of recombination-associated DNA synthesis. (a) A schematic representation of the reaction. First, RAD51 (1 μM) was loaded onto a 90 nt D1 oligonucleotide (3 μM as nucleotides) for 5 min at 37 °C. Hop2/Mnd1 complex (200 nM) was then incorporated and the mixture was incubated for 1 min at 37 °C. The reaction was started by adding pBluescript SK(−) replicative form I (50 μM as base pairs) and incubating for 5 min at 37 °C. Next, PCNA (30 nM) was loaded by RFC complex (10 nM) in the presence of RPA (666 nM) and dNTPs (100 μM each) for 5 min at 30 °C. The extension was initiated by incorporation of Pol δ (30 nM). After 10 min at 37 °C, reactions were stopped, treated with proteinase K, and then analyzed. (b) All tested proteins are required for efficient DNA repair synthesis. The reactions were performed as described above. Individual proteins were omitted as indicated. α-[^32^P]-dATP was used to follow the reactions. Labeled lambda DNA digested with BstEI was used as a marker (only a subset of bands is depicted).

In order to analyze the molecular mechanism of D-loop extension during HR in humans, we reconstituted the recombination-associated DNA synthesis in vitro by coupling the D-loop formation with DNA synthesis (Fig. 1A). RAD51 was first nucleated on D1 oligonucleotide in the presence of ATP, which was followed by incorporation of Hop2–Mnd1 mediator complex. D-loop formation was initiated by addition of the donor plasmid, as described previously [21,22]. PCNA was then loaded onto the substrate by the RFC-complex and the extension was initiated by addition of Pol δ. The reactions were separated on an agarose gel under native conditions and monitored either by following the labeled oligonucleotide or by incorporation of α-[^32^P]-dATP.

Strong incorporation of α-[^32^P]-dATP was observed only when all the aforementioned factors were present in the reaction (Fig. 1B). Some minor incorporation of α-[^32^P]-dATP was detected in the absence of RAD51 or Hop2-Mnd1 (Fig. 1B, lanes 1 and 2), indicating some unspecific incorporation due to presence of the nick in the DNA substrate. No incorporation occurred when Pol δ, PCNA or RFC were omitted (Fig. 1B, lanes 3–5). Notably, addition of the single-strand binding protein replication protein A (RPA) results in formation of longer extension tracks (Fig. 1B, lane 7). Longer extension as well as twofold increase of the extended D-loop was seen when the reaction was performed using labeled D1 oligonucleotide or yeast RPA (Supplementary Fig. 1B). Taken together, PCNA loaded onto RAD51-mediated D-loop substrate together with Pol δ are sufficient for recombination-associated DNA synthesis. Moreover, RPA stimulates the processivity of the polymerase, perhaps by preventing the formation of secondary structures and promoting strand displacement.

### 3.2 Human polymerase δ efficiently extends D-loops in vitro

**Fig. 2:**
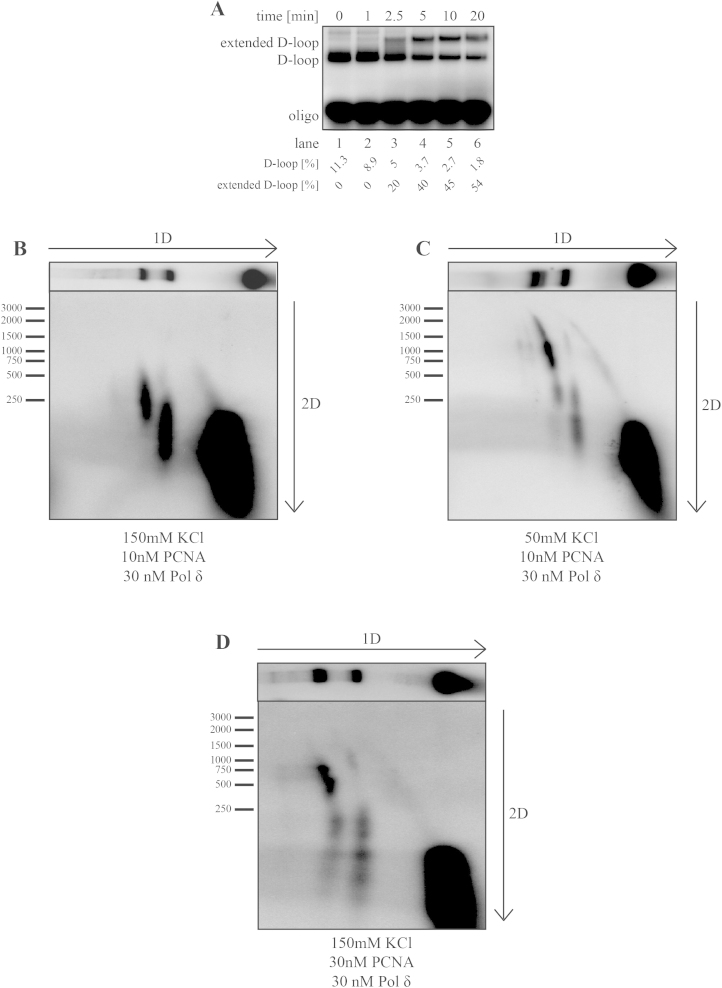
Characterization of DNA repair synthesis by Pol δ. (a) Pol δ extends up to 50% of D-loop in a time-course experiment. Reactions were performed as described in Section 2. At indicated time points after addition of Pol δ (30 nM), the samples were withdrawn and separated on agarose gel. (b) Pol δ extends up to 250 nt under suboptimal conditions. 2D-gel analysis of the length of the D-loop extension in the presence of 30 nM Pol δ, 10 nM PCNA, 150 mM KCl and 3 kb template DNA. (c) 2D gel analysis of decreasing ionic strength in the reaction results in stimulation of the extension by Pol δ. The reaction was performed as in (b) except using 50 mM KCl. (d) Stoichiometric concentration of PCNA partially suppresses the effect of ionic strength by 2D gel analysis. The reaction was performed with 30 nM PCNA, 30 nM Pol δ and 150 mM KCl. Labeled 1 kb DNA ladder was used as a marker (only a subset of bands is depicted in the figures). Labeled D1 oligonucleotide was used to monitor the reactions. The percentage [%] of D-loop was calculated as the amount of D1 oligonucleotide incorporated into the donor plasmid. Quantification [%] of extended D-loop denotes the fraction of D-loop that was extended.

To gain more insight into the kinetics of recombination-associated DNA synthesis by Pol δ, the efficiency of the reaction was determined in a time-course experiment using labeled D1 oligonucleotide. As shown in Fig. 2A, already at 2.5 min, 20% of D-loops were extended by Pol δ and more than 50% of D-loops were extended at 20 min. We then wanted to assess the length of the extension products by changing the ionic strength in the reaction and the PCNA concentration, and 2D gel electrophoresis was used to estimate the length of the extension products. In the first dimension, the extended D-loop was separated from D-loop and D1 oligonucleotide under native conditions. In the second dimension, the DNA was separated under denaturing conditions. Initially, the length of extension at sub-stoichiometric concentration of PCNA (10 nM) to Pol δ (30 nM) and 150 mM KCl reached on average 250 nt with maximum up to 500 nt (Fig. 2B). Under lower salt concentration (50 mM KCl), the length reached an average size of 1000 nt with maximum length up to 2000 nt (Fig. 2C). Increasing the PCNA concentration to 30 nM while maintaining a physiological salt concentration led to an increase in average length of extension products to around 750 nt and maximum to about 1000 nt (Fig. 2D), thus indicating that lower salt or increasing concentration of PCNA might stabilize the complex with Pol δ and increase its processivity. These data suggest that human Pol δ efficiently extends RAD51-mediated D-loops and that the in vitro extension length, which depends upon the PCNA and salt concentrations, ranges from 100 nt to 2000 nt.

### 3.3 Human TLS polymerases η and κ participate in HR in vivo

**Fig. 3:**
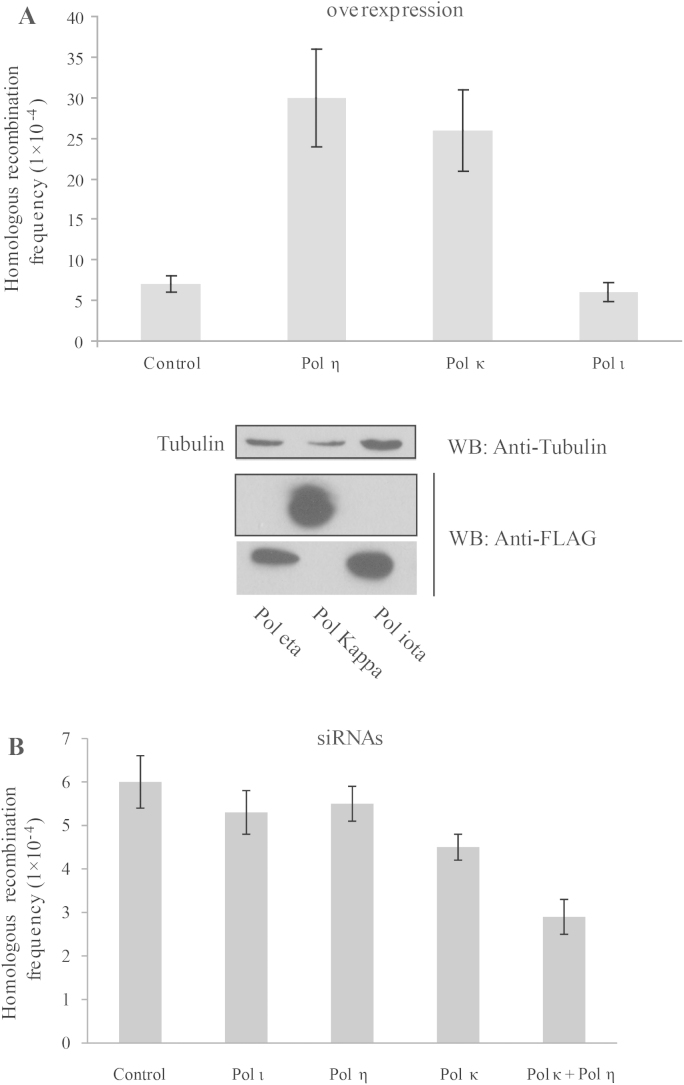
Effect of overexpression and down-regulation of Pol ι, Pol η or Pol κ on I-SceI-induced homologous recombination. Frequencies of recombination were measured from the frequency of HR-dependent restoration of GFP-positive cells after (a) the overexpression of Pol ι, Pol η or Pol κ; (b) siRNAs knockdown of Pol ι, Pol η, Pol κ; or knockdown of both Pol η and Pol κ. Error bars show standard deviation of the data obtained from three independent experiments. The expression level of the expressed proteins is indicated below panel (a).

We next tested whether any TLS polymerase might be involved in the DNA synthesis step of HR. Therefore, a modified reporter assay was applied to monitor the frequency of HR events in vivo using DSB induced by I-SceI (Supplementary Fig. 2A). This method monitors the restoration frequency of full-length GFP by recombination between the targeted construct containing a C-terminally truncated GFP and an I-SceI recognition site and the construct containing an N-terminally truncated GFP. To validate the system, we expressed or silenced several components known to influence the homologous recombination, including XPF, XRCC4, PARP1 and Rad51AP1, and then observed the phenotypes similar to ones reported previously (Supplementary Fig. 2B [23–26]). To analyze the effect of the different DNA polymerases on HR, the human HR reporter cells were transfected with a vector for overexpression of several TLS polymerases, including Pol η, Pol κ and Pol ι. Notably, overexpression of Pol η or Pol κ, respectively, resulted in 6-fold or 5-fold increase in HR frequency (Fig. 3A). On the other hand, the presence of an empty vector or overexpression of Pol ι had no effect on the frequency of HR (Fig. 3A), thus suggesting that Pol η and Pol κ, but not Pol ι, contribute to HR in humans.

To further support this conclusion, we also examined the frequency of HR after siRNA depletion of these polymerases. However, no effect on HR frequency was observed after down-regulating any of the TLS polymerases. Surprisingly, the silencing of both Pol η and Pol κ resulted in 2-fold reduction of the HR frequency (Fig. 3B), suggesting their role in the DNA synthesis step of HR and also indicating possible redundancy of these TLS polymerases in HR.

### 3.4 Human polymerase η extends D-loop substrate independently of PCNA

**Fig. 4:**
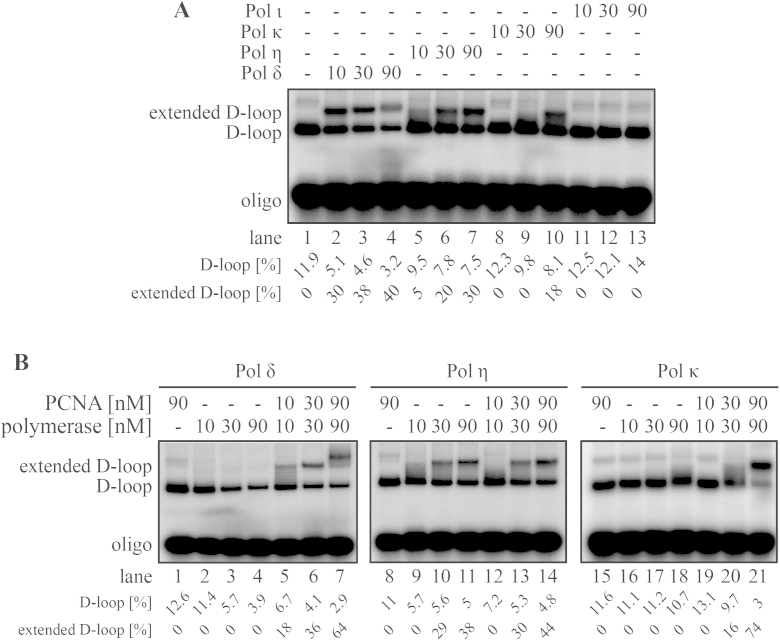
Pol η and Pol κ extend RAD51-mediated D-loops. (a) Pol η- and Pol κ-mediated D-loop extension. The reactions were carried out with increasing concentrations (10, 30, and 90 nM) of Pol δ (lanes 2–4), Pol η (lanes 5–7), Pol κ (lanes 8–10) and Pol ι (lanes 11–13), respectively. (b) The extension by Pol η is PCNA independent. Increasing concentrations (10, 30, and 90 nM) of Pol δ (lanes 2–7), Pol η (lanes 9–14) or Pol κ (lanes 15–21) were incubated in the absence (lanes 2–4, 9–11, and 16–18) or presence (lanes 5–7, 12–14, and 19–21) of equimolar PCNA concentration. Labeled D1 oligonucleotide was used to monitor the reaction. The percentage [%] of D-loop was calculated as the amount of D1 oligonucleotide incorporated into donor plasmid. Quantification [%] of extended D-loop denotes the fraction of D-loop that was extended.

To confirm the role of these members of the Y-family of TLS polymerases in HR, we tested them in the RAD51-mediated D-loop extension assay. In this reaction, 10 nM Pol δ extended 30% of the substrate within 10 min. On the other hand, 9 times more Pol η and Pol κ were necessary to reach extension of 30% and 18% of D-loops, respectively (Fig. 4A). This could not be a reflection of the enzyme's activity alone, as similar extension for Pol δ, Pol η and Pol κ was observed in a control experiment using an oligonucleotide-based substrate in the absence of PCNA (Supplementary Fig. 3A). Longer incubation time results also in more D-loop extension, but 2D gel analysis revealed incorporation of only limited numbers of nucleotides (<100 nt) by either Pol η or Pol κ (Supplementary Fig. 3B–D).

We then wanted to test the effect of PCNA on DNA extension. Increasing concentration of PCNA resulted in stimulation of D-loop extension by Pol δ or Pol κ, but it had no effect on extension by Pol η (Supplementary Fig. 3E), thus suggesting PCNA-independent synthesis in the case of Pol η. This was further supported by a similar experiment performed in a low salt concentration (50 mM KCl). Under these conditions, not only Pol η but also Pol κ start to extend the D-loops independently of PCNA (Supplementary Fig. 3E). This is in contrast to Pol δ, the extension of which is fully dependent on PCNA under all tested conditions (Fig. 4B and Supplementary Fig. 3E). Moreover, Pol η extension of the D-loop is PCNA-independent even under low protein concentration (Fig. 4B), thus indicating that it is not due to an excess of Pol η in the reaction. To further support the notion that Pol η, unlike Pol δ, works independently of PCNA, a competition experiment was conducted. We challenged the D-loop extension by both polymerases with addition of an excess of oligonucleotide-based substrate, which is extended independently of PCNA. While Pol δ extended the D-loop to the same extent in the presence of a competitor substrate, extension by Pol η was inhibited (Supplementary Fig. 4), indicating that presence of PCNA may play a role in the regulation of the polymerase choice during HR in humans.

To ascertain the in vivo relevance of the PCNA-independent D-loop extension by DNA polymerase η, the expression effects of wild-type and PCNA-binding-deficient Pol η (PIP1, PIP2 mutant) [27] were compared using our HR reporter system. We found that the Pol η PIP mutant stimulated HR with almost the same efficiency as did the wild-type Pol η (Supplementary Fig. 5), indicating that PCNA-binding is not required for Pol η function in HR.

## 4 Discussion

Homologous recombination is a crucial DNA repair pathway to maintain genome integrity through accurate repair of DSBs and ssDNA gaps left behind the replication fork. While many of the steps in HR have been studied in great detail, little is known about the recombination-associated repair synthesis and many questions remain unanswered: which polymerases and other accessory factors are required for this step? How are the individual factors recruited to the site of DNA extension, and what is their turnover after completion of DNA synthesis? What are the molecular mechanisms of DNA extension and the role of chromatin?

This study aimed to determine the essential components and conditions of recombination-associated DNA synthesis and their molecular mechanisms of action. RAD51-mediated D-loop extension with purified human proteins was reconstituted. We found that the replicative human polymerase Pol δ, together with loaded PCNA, can efficiently extend D-loops formed by RAD51 protein. This is well in accordance with findings of yeast-based genetic studies [16–18,28] as well as biochemical experiments [19,20]. Moreover, the stimulation of DNA synthesis by RPA might be explained by possible prevention of the formation of secondary structures and promotion of strand displacement, indicating that this reaction may even proceed via a rolling circle mechanism. The observed maximum length of the extension corresponds fully with the extension track length observed in mammals in vivo [29,30]. Furthermore, the effect of various conditions on the extension length can have a significant impact on the later steps of recombination. We have recently shown that regulation of the length of extension by Srs2 correlates with the increase in crossover frequency, an outcome that could be deleterious for mitotically dividing cells [31].

There also is contending evidence, however, that various TLS polymerases might be involved in HR [6–8,16,31], and we therefore wanted to test their roles during HR in human cells. Overexpression of Pol η and Pol κ resulted in 5-fold and 6-fold stimulation in HR frequency, respectively, thus suggesting their roles in repair of DSB induced by I-SceI. While their individual down-regulation did not result in an obvious change in HR frequency, simultaneous depletion of Pol η and Pol κ did result in 2-fold reduction, thus indicating their redundant roles during HR in human cells. In contrast, overexpression or down-regulation of Pol ι had no effect on recombination frequency, indicating that only some TLS polymerases might be involved in HR. The involvement of Pol κ in HR was unexpected, as chicken DT-40 and mouse cells lacking this TLS polymerase have shown no phenotype upon exposure to ionizing radiation [32,33]. This could be due to the different nature of the DNA ends generated by the ionizing radiation and I-SceI, or it could be due to the redundancy of the system, as a significant effect is observed only in combination with down-regulation of Pol η. Alternatively, the involvement of Pol κ in human cells may be more pronounced. These data indicate that various polymerases may be involved in the D-loop extension in vivo. On the basis of these in vivo observations, we characterized the effect of these polymerases on D-loop extension. Both Pol η and Pol κ extended the D-loop substrate, but they did so less efficiently than did Pol δ. This difference in efficiency may be due to the lack of strand-displacement activity by the TLS polymerases or other accessory proteins. In the case of Pol η and Pol κ, moreover, the length of the extended track reached around 150 nt, thus suggesting that these polymerases may extend shorter tracks in vivo. Notably, while Pol δ showed PCNA-dependent synthesis under all tested conditions, Pol η extended D-loop substrate independently of PCNA. Based on this biochemical finding, we analyzed the effect of mutation in the PCNA binding domain of Pol η on HR in vivo and found that, similarly to wild type, the PIP mutant Pol η was able to stimulate HR, lending further support to PCNA-independent D-loop extension by Pol η. While Pol κ also showed PCNA-independent D-loop extension, it did so only under low salt concentration. These data suggest that PCNA could affect the outcome of HR by targeting an appropriate polymerase complex to the site of the action.

**Fig. 5:**
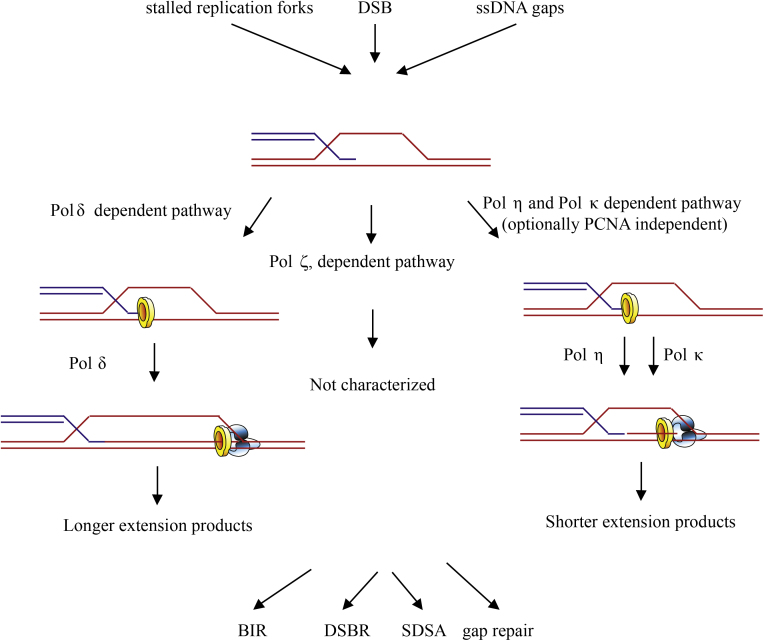
Model of regulation of the length of D-loop extension by PCNA and choice of polymerase. HR can act on diverse substrates (e.g., DSBs formed by DNA-damaging agents or ssDNA gaps left behind replication fork). Irrespective of the inputs, HR proceeds by formation of D-loop, representing a common structure formed by an invading strand of the broken DNA into homologous template sequence, in order to copy the missing information. We propose that presence of PCNA on the D-loop might target the appropriate polymerase and determine the outcome of HR by regulating the extension length. When loaded, PCNA promotes a long extension track by Pol δ. In the absence of PCNA, on the other hand, Pol η may extend the D-loop generating shorter extension tracks, which could correlate with use in the gap repair and template switch events. The length of the extension might also be important for synthesis-dependent strand-annealing (SDSA) or double-strand break repair (DSBR), which are characterized, respectively, by displacement of the extended D-loop or stabilization of D-loop by second capture. PCNA loading may thus represent a regulatory point for appropriate choice of pathway. Alternatively, break-induced replication can take place when a second end of DSB is absent.

While all those lesions processed by DSB repair lead to formation of D-loop, several alternative outcomes can follow DNA repair synthesis. Our data, together with previously published observations, point to a possible model for regulation of the D-loop extension (Fig. 5) [6–8]. In agreement with previous data, Pol δ constitutes an efficient and processive pathway responsible for the generation of longer track length. Nevertheless, the existence of several TLS polymerases indicates that several alternative pathways may exist for copying the missing information during HR. Indeed, our data suggest that Pol η and Pol κ affect recombination in vivo and can participate in D-loop extensions in vitro that result in generating short extension tracks. A third pathway for extension of the D-loop involving Pol ζ and Rev1 has also been suggested [6]. No biochemical characterization of a Pol ζ/Rev1-dependent D-loop extension has yet been described, however, although depletion of both Rev3 (the catalytic subunit of Pol ζ) and Rev1 results in decreased HR frequency [6]. Furthermore, we also suggest that PCNA might be a regulatory component that plays an active role in targeting one or more appropriate polymerases. While it is tempting to speculate that these independent extension pathways may provide the cells with a regulatory point allowing various outcomes from HR, more experiments are required to understand this regulation and its components. Finally, our reconstitution system also provides an excellent tool for studying the role of PCNA post-translational modification (e.g., ubiquitination [13] and SUMOylation [14]), as we have recently shown in the yeast system [20] by characterizing the molecular mechanism of Srs2-dependent inhibition of DNA repair synthesis [34]. Similarly, additional emerging factors that recognize these modifications in humans can be tested (e.g., Spartan [35,36], ZRANB3 [37–39], and PARI [15]).

## Conflict of interest statement

The authors declare that there is no conflict of interest.
