# Supplementary material for: Role of PCNA and TLS polymerases in D-loop extension during homologous recombination in humans
Source: DNA Repair (Amst). 2013 Sep;12(9):691–8. doi: 10.1016/j.dnarep.2013.05.001 (PMC3744802; doi:10.1016/j.dnarep.2013.05.001)

# SUPPLEMENTARY INFORMATION TO THE MANUSCRIPT:

# Role of PCNA and TLS polymerases in D-loop extension during homologous recombination in humans

Marek Sebesta[a,b,c], Peter Burkovics[b,d], Szilvia Juhasz[d], Sufang Zhang[e], Judit E. Szabo[a,f], Marietta Y.W.T. Lee[e], Lajos Haracska[d], Lumir Krejci[a,b,c*]

[a]National Centre for Biomolecular Research and [b]Department of Biology, Masaryk University, Brno, Czech Republic. [c]International Clinical Research Center, Center for Biomolecular and Cellular Engineering, St. Anne's University Hospital Brno, Brno, Czech Republic. [d]Institute of Genetics, Biological Research Center, Hungarian Academy of Sciences, Szeged, Hungary. [e]Department of Biochemistry and Molecular Biology, New York Medical College, Valhalla, NY, USA. [f]Institute of Enzymology, Biological Research Center, Hungarian Academy of Sciences, Budapest, Hungary.

CONTENTS:

Supplementary Material and Methods

Supplementary Figure Legends

Supplementary Figures 1-5

## SUPPLEMENTARY MATERIALS AND METHODS

### *DNA substrates*

Oligonucleotides were purchased from VBC Biotech and corresponding substrates were prepared as in [1].

***Plasmids***

pBluescript SK replicative form I was purified using NucleoBond® Xtra Midi kit from Macherey-Nagel according to the manufacturer's instructions. Flag-Pol η PIP mutant containing the F443A, L444A, F707A, F708A was generated by side-directed mutagenesis of pRK2F plasmid [2]. The construct was verified by sequencing.

***Protein purifications***

*PCNA purification* - Human PCNA was expressed in *E. coli* BL21 RIPL cells (induction: 37ºC, 1mM IPTG, 3 h) as a GST-PCNA fusion protein with a Pressicion-Protease cleavage site between GST and PCNA. All purification steps were performed at 4ºC. Ten grams of *E. coli* cell paste was sonicated in 50 ml of lysis buffer C (50 mM Tris-HCl (pH 7.5), 10 % sucrose (w/v), the protease inhibitors (aprotinin, chymostatin, leupeptin, pepstatin A, benzamidine, each at 5µg/ml), 10 mM EDTA, 1mM dithiothreitol (DTT), 0.01% v/v Nonidet-P40, and 100 mM KCl. The crude lysate was clarified by centrifugation (100,000xg for 90 min). The supernatant was loaded onto a 7-ml Q sepharose column (GE Healthcare) equilibrated with buffer T (25 mM Tris-Cl, 10 % (v/v) glycerol, 5 mM EDTA, pH 7.5) containing 100 mM KCl, and eluted with a 110 ml linear gradient of 100 - 900 mM KCl in buffer T. The peak fractions were pooled, and batched for 2 h with 2 ml of GTH-agarose (GE Healthcare Life Sciences) equilibrated with buffer T containing 100 mM KCl. PCNA was eluted by over-night incubation with Pressicion-Protease (5 µg) in 2 ml of buffer T with 100 mM KCl. After the elution, the beads were washed five times with 2 ml of buffer T containing 100 mM KCl. Fractions containing cleaved PCNA were pooled, diluted with 5 ml of buffer T, and loaded onto a 1-ml Mono Q column (GE Healthcare). PCNA was eluted with a 15-ml gradient of 100 - 900 mM KCl in buffer T. Fractions containing homogenous PCNA were concentrated in a Vivaspin concentrator (Sartorius Stedim Biotech), and stored in a 5-µl aliquots at −80°C.

*RPA purification* - Untagged human RPA was expressed in *E.coli* Rosetta (DE3) cells (induction: 37ºC, 0.5 mM IPTG, 3 h) from pLK966 plasmid, which was originally described in [3] and kindly provided by Patrick Sung. All purification steps were performed at 4ºC. Twenty grams of *E. coli* cell paste was sonicated in 100 ml of lysis buffer C, containing 100 mM KCl. The crude lysate was clarified by centrifugation (100,000xg for 90 min). The supernatant was loaded onto a 20-ml Affi-Blue column (BioRad) equilibrated with buffer T containing 100 mM KCl, washed with 80 ml of buffer T containing 100 mM KCl and followed by elution with a 80-ml linear gradient of 750 - 2500 mM NaSCN in buffer T. The peak fractions were pooled and loaded onto a 5-ml hydroxyapatite column (BioRad) equilibrated with buffer T containing 100 mM KCl. After the wash with 50 ml of buffer T supplemented with 100 mM KCl, proteins were eluted with a 50-ml linear gradient of 20-220 mM $KH_2PO_4$. Fractions containing RPA were pooled, diluted with 10 ml of buffer T and loaded onto a 1-ml Mono Q column (GE Healthcare Life Sciences) equilibrated with buffer T containing 100 mM KCl. Proteins were eluted with a 20-ml gradient of 100 - 900 mM KCl in buffer T. Fractions containing nearly homogenous RPA complex were concentrated in a Vivaspin concentrator and stored in a 10 µl aliquots at −80°C.

*RAD51 purification* - untagged human RAD51 was expressed in *E.coli* BLR (DE3) pLysS cells (induction: 37ºC, 1mM IPTG, 3 h), from pET11-RAD51 plasmid described elsewhere [4]. All purification steps were performed at 4ºC. Sixty grams of *E. coli* cell paste was sonicated in 300 ml of lysis buffer C containing 1 M KCl. The crude lysate was clarified by centrifugation (100,000xg for 90 min). Proteins were precipitated with $(NH_4)_2SO_4$ (0.242 g/ml) and spun (10,000xg for 20 min). The pellet was resuspended in 150 ml of buffer K (20 mM $K_2HPO_4$, 10 % (v/v) glycerol, 0.5 mM EDTA, 0.01 % (v/v) NP40, and 1 mM DTT, pH 7.5) and loaded on 40-ml Q sepharose column equilibrated with buffer K containing 175 mM KCl. Proteins were eluted with a 400-ml linear gradient of 175-500 mM KCl in buffer K. The peak fractions were pooled and loaded onto a 5-ml

hydroxyapatite column equilibrated with buffer T containing 100 mM KCl. After a wash with 50 ml of buffer T containing 100 mM KCl, proteins were eluted with a 100-ml linear gradient of 60-260 mM $KH_2PO_4$. Fractions containing RAD51 were pooled, diluted with 20 ml of buffer T, loaded onto a 1-ml Mono Q column equilibrated with buffer T containing 100 mM KCl and eluted with a 60-ml gradient of 100 - 500 mM KCl in buffer T. Fractions containing nearly homogenous RAD51 were concentrated in a Vivaspin concentrator and stored in a 10 µl aliquots at −80°C.

*Purification of other proteins* - Pol δ was purified according to previously published procedure in [5]. Pol η, Pol κ, Pol ι were expressed in yeast as GST-fussed proteins and purified according to previously described procedure [6]. RFC complex, Hop2-Mnd1 complex and yRPA were purified as described elsewhere [7-9], respectively.

***Oligo-based extension assay***

The fluorescently labeled 5' overhang (600 nM) was incubated with either 10 nM Pol δ, Pol η, Pol κ or Pol ι in buffer B (25 mM Tris-Cl pH 7.5, 0.2 mg/ml BSA, 1 mM DTT, 60 mM KCl, 8 mM $MgCl_2$ 100 µM dNTPs). Samples were withdrawn at 0, 1, 2.5, 5 and 10 min after addition of the corresponding polymerase. The reaction was stopped with 5 min incubation at 37ºC with Proteinase K (to 1 µg/µl). Samples were boiled for 5 min in an equal volume of loading buffer (90 % (v/v) formamide, 0.002 % (w/v) Orange C) and resolved on 13 % denaturing PAGE gel. After electrophoresis, fluorescent DNA species were visualised in a Fuji FLA 9000 imager and analysed with Multi Gauge software (Fuji).

***Competition assay***

The reactions were performed essentially as described for the primer-extension assay except the buffer B (instead of buffer O) contained 50 mM KCl. First, PCNA was loaded on D-loops, followed

by addition of Pol δ or Pol η. The competing substrate (5‘overhang, 40 nM, fluorescently labeled) was added together with dATP and dTTP, which served to start the reactions. In control experiments, either D1 oligo was omitted or 5’ overhang was omitted form the mixture and reactions were separate on 0.8% agarose gel (to visualise the extension from the D-loop substrate) and/or 13% denaturing PAGE gel (to visualise the oligo-based DNA extension). After the separation, the agarose gel was dried, exposed on screen, and visualised in a Fuji FLA 9000 imager and analysed with Multi Gauge software (Fuji). While the fluorescent DNA species were visualised in a Fuji FLA 9000 imager and analysed with Multi Gauge software (Fuji).

## REFERENCES

[1] Matulova,P., Marini,V., Burgess,R.C., Sisakova,A., Kwon,Y., Rothstein,R., Sung,P. and Krejci,L. Cooperativity of Mus81·Mms4 with Rad54 in the Resolution of Recombination and Replication Intermediates. Journal of Biological Chemistry **284** (2009) 7733–7745.

[2] Acharya,N., Yoon,J.-H., Gali,H., Unk,I., Haracska,L., Johnson,R.E., Hurwitz,J., Prakash,L. and Prakash,S. Roles of PCNA-binding and ubiquitin-binding domains in human DNA polymerase η in translesion DNA synthesis. Proceedings of the National Academy of Sciences **105** (2008) 17724–17729.

[3] Henricksen,L.A., Umbricht,C.B. and Wold,M.S. Recombinant replication protein A: expression, complex formation, and functional characterization. Journal of Biological Chemistry **269** (1994) 11121–11132.

[4] Sigurdsson,S., Trujillo,K., Song,B., Stratton,S. and Sung,P. Basis for Avid Homologous DNA Strand Exchange by Human Rad51 and RPA. Journal of Biological Chemistry **276** (2001) 8798–8806.

[5] Meng,X., Zhou,Y., Zhang,S., Lee,E.Y.C., Frick,D.N. and Lee,M.Y.W.T. DNA damage alters DNA polymerase  to a form that exhibits increased discrimination against modified template bases

and mismatched primers. Nucleic Acids Research **37** (2008) 647–657.

[6] Gali,H., Juhasz,S., Morocz,M., Hajdú,I., Fatyol,K., Szukacsov,V., Burkovics,P. and Haracska,L. Role of SUMO modification of human PCNA at stalled replication fork. Nucleic Acids Research **40** (2012) 6049–6059.

[7] Pezza,R.J., Petukhova,G.V., Ghirlando,R. and Camerini-Otero,R.D. Molecular Activities of Meiosis-specific Proteins Hop2, Mnd1, and the Hop2-Mnd1 Complex. Journal of Biological Chemistry **281** (2006) 18426–18434.

[8] Finkelstein,J., Antony,E., Hingorani,M.M. and O'Donnell,M. Overproduction and analysis of eukaryotic multiprotein complexes in Escherichia coli using a dual-vector strategy. Analytical Biochemistry **319** (2004) 78–87.

[9] Van Komen,S., Macris,M., Sehorn,M.G. and Sung,P. Purification and Assays of Saccharomyces cerevisiae Homologous Recombination Proteins. In Judith Campbell,A.P.M. (ed), Methods in Enzymology **408** (2006) 445–463.

## SUPPLEMENTARY FIGURES

**Supplementary Figure 1. A)** Proteins used in the study were separated on a 15 % SDS-PAGE and stained with Coomassie Blue. **B)** D-loop extension reactions were performed with increasing concentrations (6.6, 66, 666 nM) of human (lanes 3-5) or yeast RPA (lanes 6-7), respectively. Labeled 1 kb DNA ladder was used as a marker (only a subset of bands is depicted). Labeled D1 oligo was used to monitor the reactions. The proportion [%] of D-loop was calculated as the amount of D1 oligonucleotide incorporated into donor plasmid. Quantification [%] of extended D-loop denotes the fraction of D-loop that was extended.

**Supplementary Figure 2. A)** Schematics of the *in vivo* recombination assay. The C-terminally truncated version of GFP, containing the cleavage site for I-SceI nuclease, is integrated in the

genome. The second, N-terminally truncated, GFP copy is present on a plasmid. Upon induction of I-SceI, a gene conversion event yields a functional GFP copy. **B)** Recombination frequencies were measured in cells expressing cDNA for XPF or XRCC4 proteins, and siRNAs for knockdown of PARP1 or Rad51AP1. Error bars show standard deviation of the data obtained from three independent experiments. **(c)** Efficiency of the siRNA silencing of Pol η, Pol κ, and Pol ι.

**Supplementary Figure 3.** Characterisation of recombination-associated DNA synthesis by TLS polymerases. **A)** All polymerases efficiently extend 5' overhang substrate in a time-course experiment. A primer extension assay was performed with 10 nM Pol δ (lanes 1-4), Pol η (lanes 5-8), Pol κ (lanes 9-12) or Pol ι (lanes 13-16), respectively. At indicated times the aliquots were withdrawn and analysed on a 12% denaturing gel. The reactions were monitored using fluorescently labeled substrate. **B)** Pol η and Pol κ extend up to 40% and 20% of D-loop, respectively. Samples of the D-loop extension reaction were withdrawn at indicated time points after Pol η (90 nM; lanes 1-6) or Pol κ (90 nM; lanes 7-12) incorporation and separated on an agarose gel. Labeled D1 oligo was used to monitor the reactions. **C)** and **D)** The length of extension products by Pol η and Pol κ. The reactions were performed as described previously with 90 nM Pol η **C)** or 90 nM Pol κ **D)** and 30 nM PCNA in the presence of 150 mM KCl. Labeled 1 kb DNA ladder was used as a marker (only a subset of bands is depicted on the figure). Labeled D1 oligo was used to monitor the reactions. **E)** Pol κ extends D-loop substrate independently of PCNA in the presence of 50 mM KCl. Increasing concentration of PCNA (3, 10, 30 nM), Pol δ (30 nM; lanes 2-5), Pol η (90 nM; lanes 7-10) or Pol κ (90 nM; lanes 12-15) were incubated in the presence of 50 mM KCl for 10 min (Pol δ) or 20 min (Pol η, Pol κ). Similar set of experiments was performed in the presence of 150 mM KCl (lanes 16-30). The proportion [%] of D-loop was calculated as the amount of D1 oligonucleotide incorporated into donor plasmid. Quantification [%] of extended D-loop denotes the fraction of D-loop that was extended.

**Supplementary Figure 4.** Preferential extension of PCNA-containing substrate. **A-D)** Extension experiments with Pol δ. A time course experiment was performed with Pol δ (30 nM) and PCNA (30 nM) in the presence of radioactively labeled D-loop (PCNA dependent) and fluorescently labeled 5‘overhang substrate (PCNA independent). At indicated times, samples were withdrawn and analyzed on agarose gel **(A)** and on a denaturing acrylamide gel **(C)** to monitor extension of D-loop or oligo-based substrates, respectively. Control experiment performed as in **(A)** except only D-loop substrate **(B)** or oligo-based substrate **(D)** was used. **E-H)** Extension experiments with Pol η. A time course experiment was performed in the presence of Pol η (30 nM), PCNA (30 nM) and in the presence of radioactively labeled D-loop and fluorescently labeled 5‘overhang substrate. At indicated times, aliquots were withdrawn and analyzed on agarose gel **(E)** and on a denaturing acrylamide gel **(G)** to monitor D-loop extension or oligo-based synthesis, respectively. Control experiment performed as in **(E)** except only D-loop substrate **(F)** or oligo-based substrate **(H)** was used. The proportion [%] of D-loop was calculated as the amount of D1 oligonucleotide incorporated into donor plasmid. Quantification [%] of extended D-loop denotes the fraction of D-loop being extended. Relative extensions were calculated as the proportion of completely extended oligo at given time point compared to the extension at 20 (Pol δ) or 40 (Pol η) minute.

**Supplementary Figure 5.** Effect of PCNA binding-deficient Pol η on recombination. Frequencies of recombination was measured from GFP-positive cells after the expression of Pol η or Pol η PIP1, PIP2 mutant. The levels of the expressed proteins are indicated. Error bars show standard deviation of the data obtained from three independent experiments.

# Supplementary Figure 1

**A**

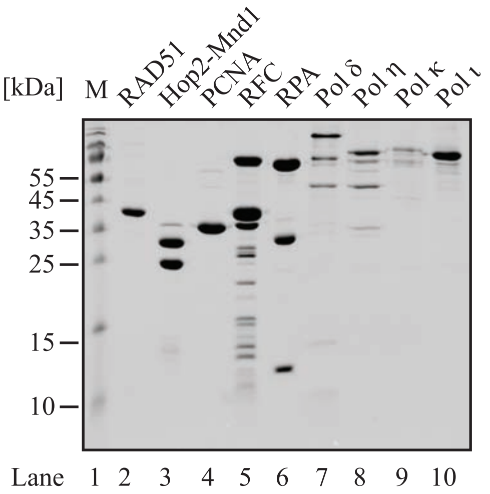


**B**

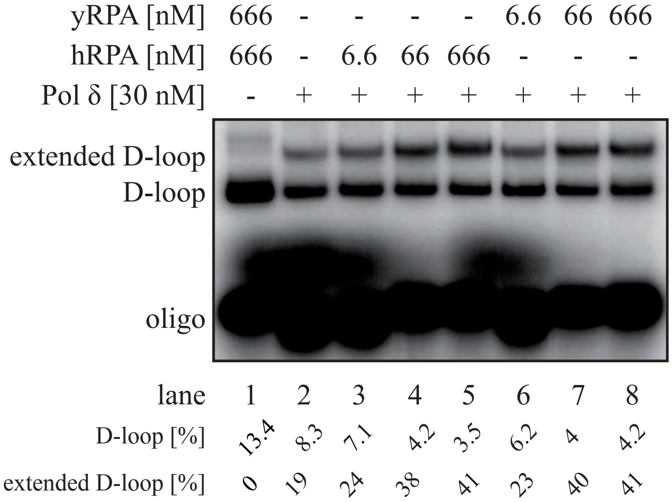

# Supplementary Figure 2

**A**

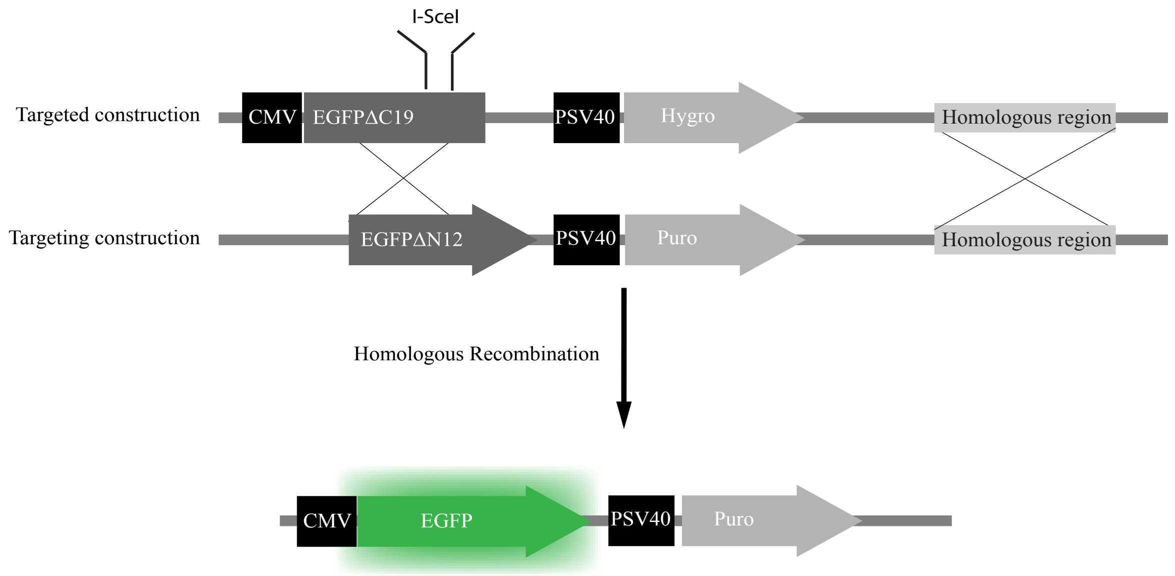


**B**

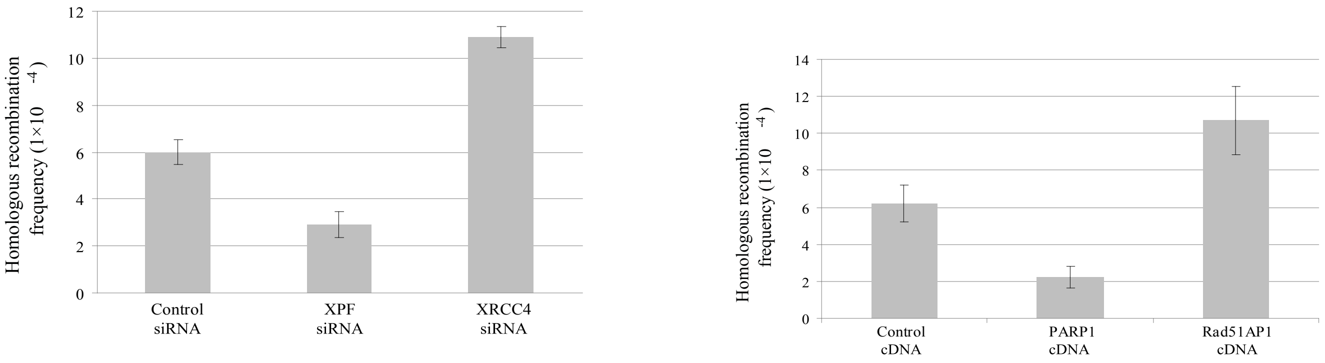


**C**

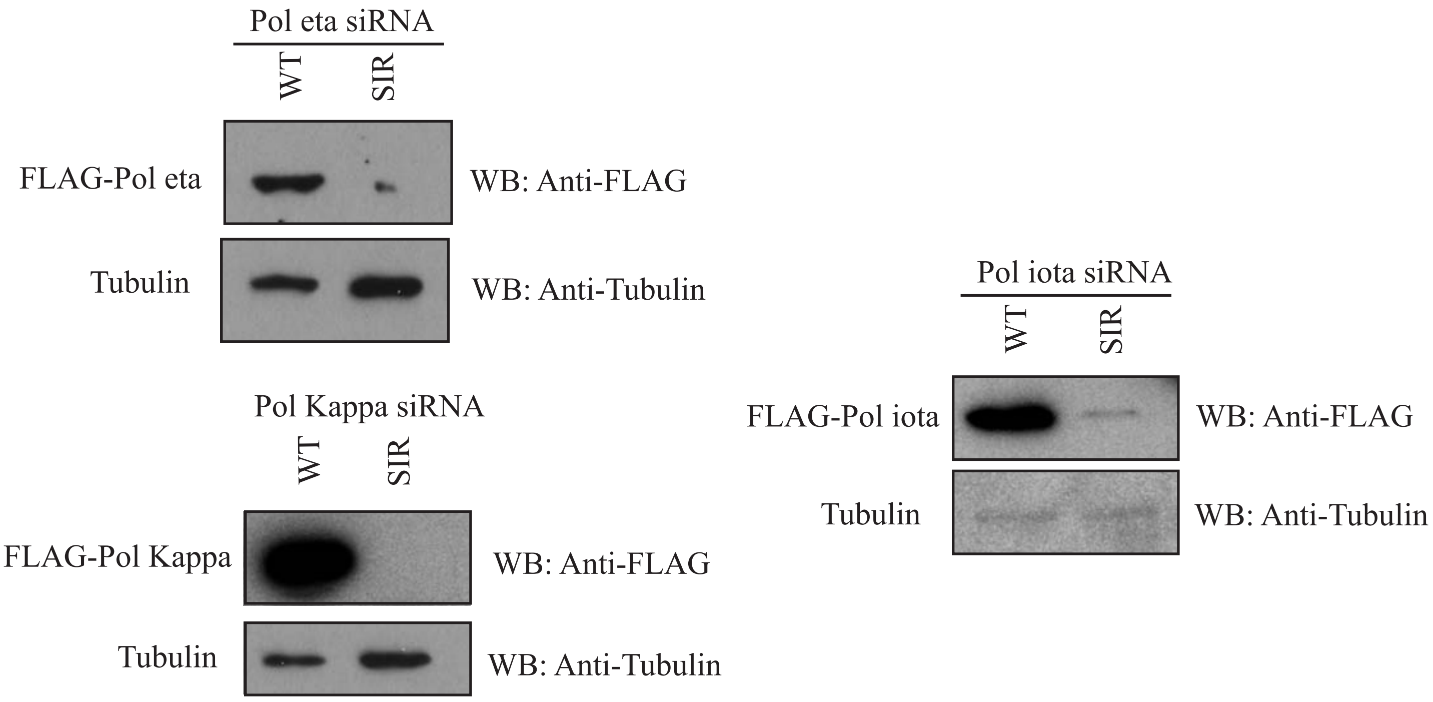

*Sebesta_et al*

**Supplementary Figure 3**

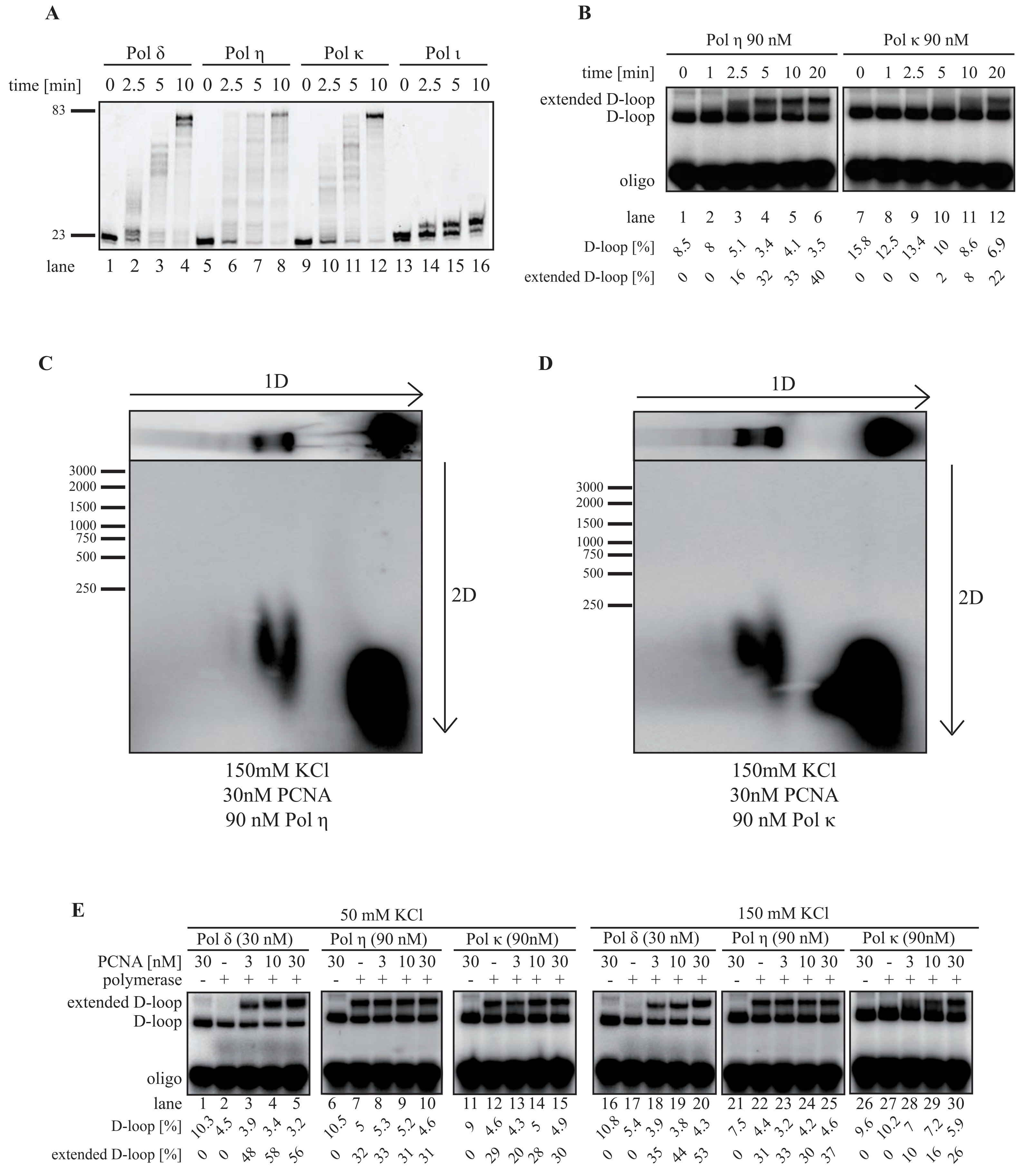

## Supplementary Figure 4

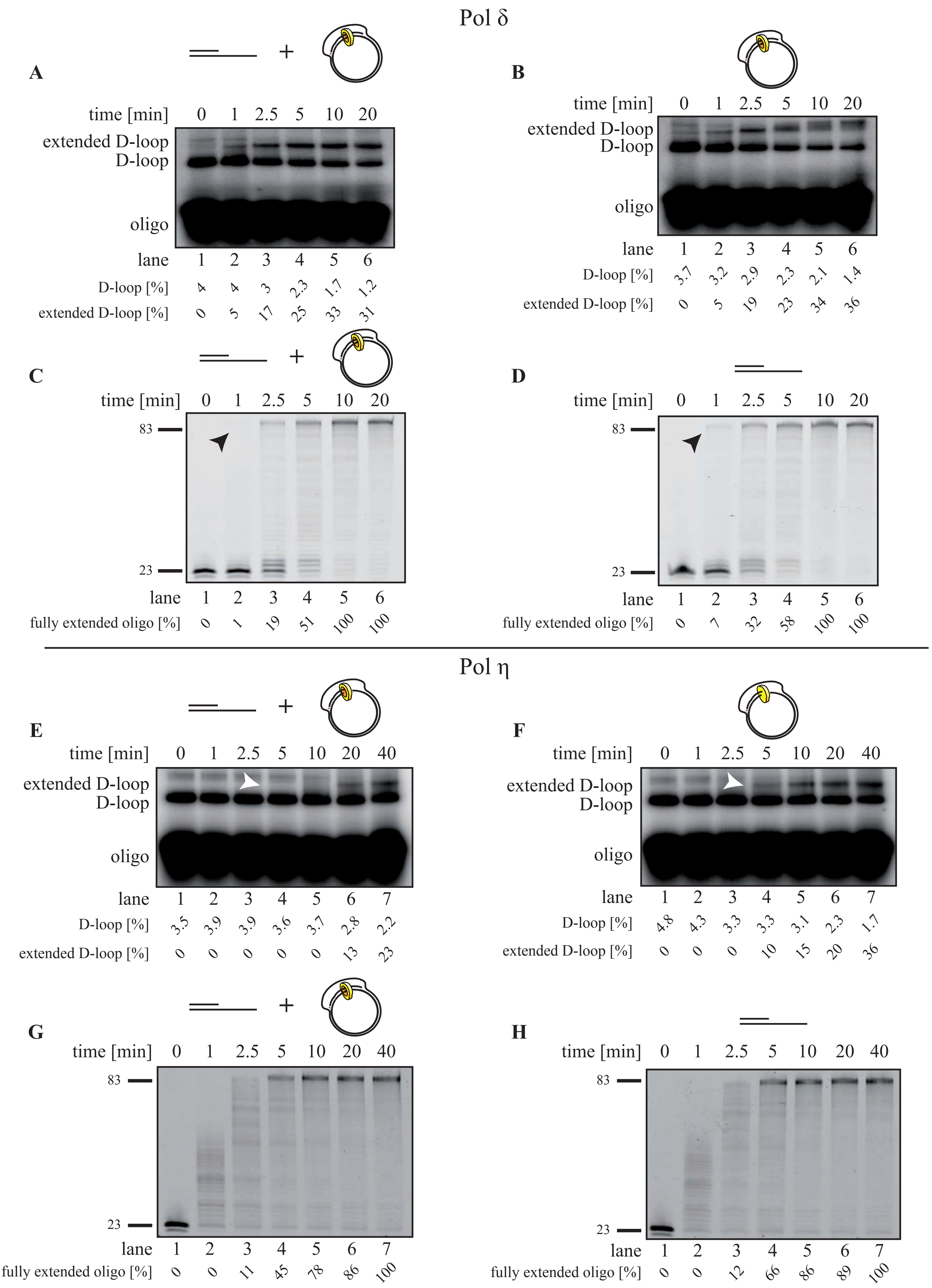

# Supplementary Figure 5

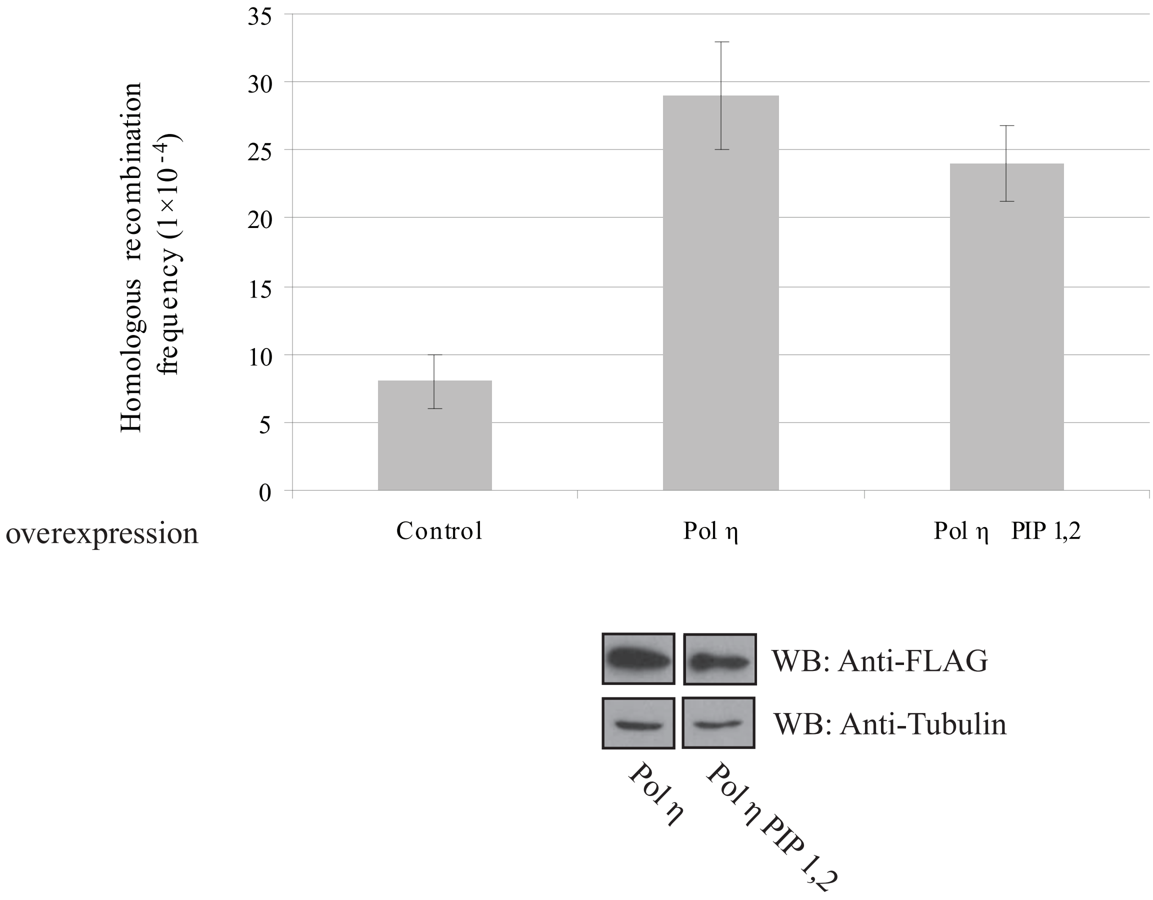

Supplement: Supplementary file 1 [file mmc1.pdf]
